# Treatment of *Helicobacter pylori* infection 14‐day concomitant quadruple therapy versus triple therapy: A parallel double‐blind randomized controlled trial

**DOI:** 10.1002/hsr2.1593

**Published:** 2023-10-04

**Authors:** Mohamed Hichem Loghmari, Firas Aissaoui, Arwa Guediche, Wided Bouhlel, Mejda Zakhama, Nabil B. Chaabene, Amel Rehaiem, Nouha Ben Abdeljalil, Manel Njima, Abdelfetteh Zakhama, Yosr Kadri, Maha Mastouri, Leila Safer

**Affiliations:** ^1^ Department of Hepato‐Gastroenterology Fattouma Bourguiba University Hospital Monastir Tunisia; ^2^ Department of Pathological Anatomy Fattouma Bourguiba University Hospital Monastir Tunisia; ^3^ Microbiology Laboratory Fattouma Bourguiba University Hospital Monastir Tunisia

**Keywords:** breath test, eradication, *Helicobacter pylori*, quadruple concomitant therapy, triple therapy

## Abstract

**Background and Aims:**

Successful *Helicobacter pylori* (Hp) eradication with the traditional 7‐day course of proton pump inhibitor triple therapy is declining. Prolonging therapy to 14 days is associated with better eradication rates. Most learned societies recommend concomitant quadruple therapy (QC) as a first‐line alternative therapy for this bacterial infection. The aim of this study is to compare the efficacy and safety of triple therapy (TT) and QC for the eradication of Hp infection.

**Methods:**

A parallel double‐blind randomized controlled trial was conducted. The diagnosis of Hp infection was made by pathological examination of gastric biopsies. Patients were randomly assigned to two treatment groups: either QC (esomeprazole 80 mg, amoxicillin 2000 mg, clarithromycin 1000 mg, and metronidazole 1000 mg daily) or triple therapy (esomeprazole 80 mg, amoxicillin 2000 mg, and clarithromycin 1000 mg daily in divided doses) for 14 days. The efficacy of the treatment is defined by Hp eradication attested by a negative breath test performed 6 weeks after the completion of treatment. Treatment outcomes were compared using the chi‐square test, while binary logistic regression identified predictors of treatment failure.

**Results:**

Ninety‐two patients were included. Forty‐two patients belonged to the QC group and 50 to the TT group. No significant difference was noted between the two groups concerning the rate of Hp eradication either by intention to treat (81% vs. 72% respectively, *p* = 0.31) or per protocol (81.6% vs. 76.1% respectively, *p* = 0.54). Likewise, there was no difference between the two groups in terms of tolerance to treatment (59.5% for QC vs. 58% for TT, *p* = 0.88). No factor has been associated with treatment failure.

**Conclusion:**

There was no significant difference in the rate of HP eradication between the QC and the 14‐day triple therapy. Neither regimen should be used topically because of their low eradication rates.

## 1 INTRODUCTION

*Helicobacter pylori* (Hp) infection is one of the most common chronic bacterial infections worldwide. In Tunisia, the seroprevalence of Hp has been estimated at 64% among blood donors^1^ and at 51.4% among children attending their first year of primary school.^2^

The involvement of Hp in the occurrence of several pathologies of the gastroduodenal mucosa, ranging from chronic gastritis to more serious conditions such as gastric adenocarcinoma and lymphoma of mucosa associated lymphoid tissue (MALT),^3^ as well as in the pathogenesis of several extra‐digestive pathologies, justifies the initiation of treatment when it is demonstrated.

According to current guidelines, the ideal antimicrobial therapy for Hp infection should have an eradication rate of at least 90%.^4^

The results of Hp eradication rates depend primarily on the rates of Hp primary resistance to clarithromycin and metronidazole, which are specific to each country.^5^, ^6^, ^7^

Given the increasing rates of resistance of *Helicobacter pylori* to triple therapy, the majority of learned societies around the world currently recommend quadruple therapy as a first‐line treatment.^4^, ^8^

The former Tunisian consensus on the treatment of Hp infection, in 2006, has not been updated. Thus, gastroenterologists tend to follow European recommendations by widely prescribing concomitant 14‐day quadruple therapies. Given the high resistance to metronidazole in Tunisia, it is not possible to know if the same combination could be as satisfactory without metronidazole. Indeed, it is recommended, when using triple therapy, to extend the treatment to 14 days because prolonging its duration increases its effectiveness.^4^ However, there is no study in Tunisia that compares triple therapy to quadruple therapy. The objective of this study is to compare the efficacy and safety of two protocols in the first‐line treatment of Hp infection in Tunisian adults: the 14‐day clarithromycin‐based triple therapy and the 14‐day concomitant quadruple therapy (QC).

## 2 MATERIALS AND METHODS

### 2.1 Context and study population

A 13‐month, parallel double‐blind, randomized controlled clinical trial was conducted from February 2019 to February 2020 in the hepato‐gastroenterology department of Fattouma Bourguiba Hospital in Monastir, Tunisia.

This study includes male and female patients aged between 18 and 65 with a documented Hp infection. The diagnosis of Hp infection was made through an anatomopathological study.^9^ Gastric biopsies were taken according to the Sydney protocol: two fundics in one pot, two antrals, and one at the angle of the lesser curve in a second pot.^10^ The biopsies were fixed in formalin and then sent to the pathology department of Fattouma Bourguiba Monastir Hospital where they were studied by an experienced anatomopathologist.

All patients have been informed in advance and have given informed consent to join the study.

The study has excluded patients:

− With cirrhosis.
− With renal failure.
− Having complicated peptic ulcer (stenosis or hemorrhage or perforation) in an acute phase.
− Having severe psychiatric disorders.
− Having history of gastric surgery.
− Having already received an Hp eradication treatment.
− Having received an antibiotic within the last 2 weeks.
− Who are allergic to one of the antibiotics used in the anti‐Hp cure.
− Who are drug addicted.

Also, pregnant or breastfeeding women were not included.

The included patients were randomly divided into two treatment groups according to a 1:1 ratio: the first group received concomitant quadruple therapy (QC) combining a double dose proton‐pump inhibitors (PPI) (esomeprazole: 40 mg × 2 per day) with amoxicillin (1 g × 2 per day), metronidazole (500 mg × 2 per day), and clarithromycin (500 mg × 2 per day) for 14 days. The second group received triple therapy (TT) combining a double dose PPI (esomeprazole: 40 mg × 2 per day) with amoxicillin (1 g × 2 per day) and clarithromycin (500 mg × 2 per day) for 14 days.

The randomization was carried out through a platform that is available online (https://secure.dacimasoftware.net/medis/View/Login.aspx) and that allows the giving of a treatment box number to every randomized patient. The treatment boxes were prepared in advance by a doctor who had a table assigning each box number to its corresponding therapy (QC or TT). To preserve the double‐blind nature of the study, two measures were essential: placing a placebo which has the same visual characteristics as metronidazole, in the boxes of the triple therapy and having a second doctor responsible for providing and explaining the treatment to patients.

### 2.2 Judging criteria

The main aim of the study was to assess the efficacy of the two treatment regimens. To achieve this goal, a breath Test was performed with a minimum time period of 6 weeks after the end of treatment. The negativity of the breath Test signals the success of the eradication.

To assess the safety of used therapies, adverse events have been carefully researched. The tolerance of the treatment is judged on the absence of adverse events or the occurrence of mild adverse events that do not affect compliance with the treatment.

To assess treatment compliance, patients were instructed to return all unused medications. A pill intake of more than 90% was considered good compliance with the treatment.^11^

### 2.3 Course of the study

**Figure 1:**
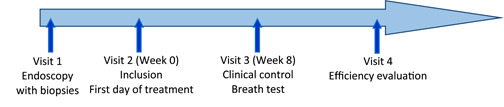
Course of the study.

After approval, the patient was informed of the progress of the study and of the need to attend scheduled visits. Figure 1 illustrates the schematization of the course of the study.

### 2.4 Sample size

The sample size was calculated using a sample size calculator (BiostatTGV). A dropout rate of 20% was considered, and the sample size was chosen to detect a 20% difference in proportions of participants who achieved Hp eradication in both treatment arms using the Pearson's chi‑squared test assuming Hp eradication rate of 70% among triple therapy group^12^ versus 90% among quadruple therapy group.^4^ These calculations were made based on the assumption that study power (1 − *β*) is 80%, and type I error (alpha) probability is 0.05. One‐sided binomial proportion test was used, so we obtained an estimated minimum sample size of 92 participants. We estimated the rate of loss of follow‐up at 20%, which makes the number of participants to be recruited at around 111.

### 2.5 Statistical study

Data were captured and analyzed using IBM Statistical Package for the Social Sciences (SPSS) version 21.0 software. The descriptive study involved a calculation of simple frequencies and relative frequencies (percentages) for the qualitative variables and a calculation of means and standard deviations for the quantitative variables or medians with interquartile ranges if the normality was not assumed (the normality was assessed by the Kolmogorov–Smirnov test).

The analytical study included the intention‐to‐treat (ITT) analysis which consists of analyzing all the patients in the group where they were randomized and the per‐protocol analysis (PP), which consists of analyzing a subgroup of the population, including only patients in full compliance with the protocol For the comparison of the percentages, the Pearson's *χ* ^2^ test was used or the Fisher test when appropriate. For the means, the Student's *t*‐test was used.

The significance level was set at *p* < 0.05 for all statistical tests. Variables significant at 20% in univariate analysis were introduced into binary logistic regression for a multivariate analysis.

### 2.6 Ethics approval

This protocol was carried out in accordance with the Declaration of Helsinki on good clinical practice after informed and signed consent by all participants before their inclusion in the protocol.

This project was exposed and approved by the Committee for the Protection of Persons of the University of Monastir‐Tunisia on February 19, 2019 with the following registration number: TN2018‐INT‐INS‐14.

## 3 RESULTS

**Figure 2:**
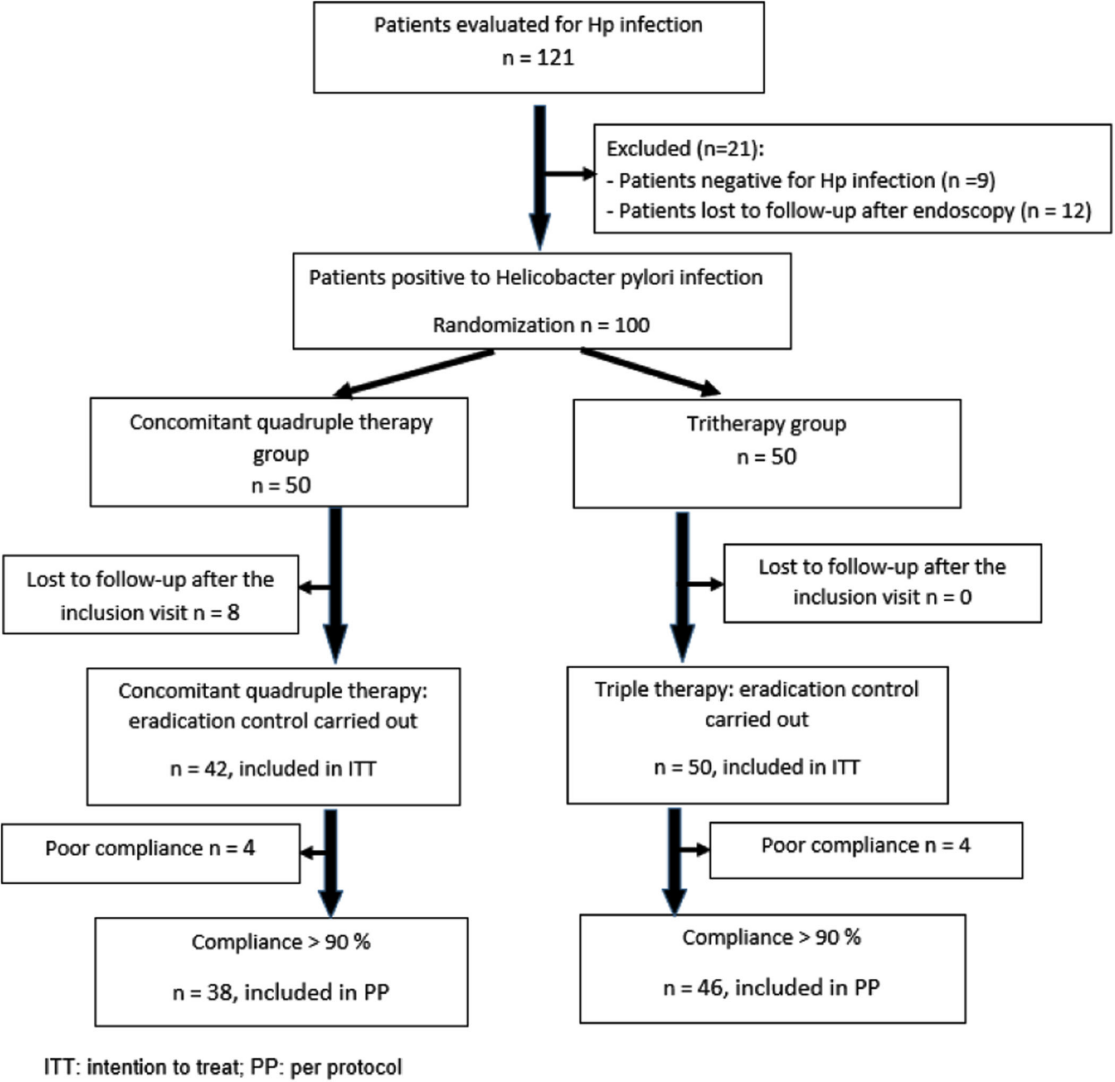
Flow diagram of the study. A total of 121 patients participated in the study, of which 100 were included in the analysis. ITT, intention to treat; PP, per‐protocol.

As shown in the flow diagram (Figure 2), a total of 121 patients were screened for Hp infection. Out of 100 patients, 50 and 50 were randomized into triple and concomitant therapies, respectively. Eight patients in the triple therapy group were lost to follow‐up.

Sociodemographic characteristics and lifestyle habits were comparable between the QC and TT groups (Table 1).

**Table 1:** Demographic characteristics of patients in the two groups.

Parameters		QC Group *N* = 42, *n* (%)	TT Group *N* = 50, *n* (%)	*p*
Age (M/SD)		41.86 ± 18.3 years	40.8 ± 12.49 years	0.7
Gender ratio [male/female]		11/31	11/39	0.64
Comorbidities	Yes	11 (26.2)	11 (22)	0.64
	No	31 (73.8)	39 (78)	
Smoking	Yes	7 (16.7)	8 (16)	0.93
	No	35 (83.3)	42 (84)	
Ethylism	Yes	0 (0)	1 (2)	>0.99
	No	42 (100)	49 (98)	
Education level	Low level	13 (30.9)	16 (32)	0.23
	High level	29 (69.1)	34 (68)	0.33
Geographic region	Urban area	35 (83.3)	40 (80)	0.68
	Rural area	7 (16.7)	10 (20)	
Average BMI [kg/m²] (M/SD)		26.89 ± 5.5	26.21 ± 4.62	0.52
Overweight (BMI ≥ 25)	Yes	27 (64.3%)	27 (54%)	0.31
	No	15 (35.7%)	23 (46%)	

Abbreviations: BMI, body mass index; M, mean; QC, concomitant quadruple therapy; SD, standard deviation; TT, triple therapy.

Endoscopic findings, dominated by erythematous gastropathy, were comparable between patients who received QC and those who received TT (Table 2).

**Table 2:** Endoscopic characteristics of patients in the two groups.

Endoscopic lesions	QC *n* (%)	TT *n* (%)	*p*
Erythematous gastropathy	39 (92.9)	39 (78)	0.07
Nodular gastropathy	2 (4.76)	9 (18)	0.06
Fundal atrophy	1 (2.38)	1 (2)	>0.99
Gastric ulcer	0 (0)	1(2)	>0.99
Duodenal ulcer	0 (0)	3 (6)	0.24
Ulcerated bulbitis	2 (4.8)	2 (4)	>0.99
Erythematous bulbitis	6 (14.3)	5 (10)	0.52
Hiatal hernia	4 (9.5)	9 (18)	0.36
Peptic esophagitis	0 (0)	1 (2)	>0.99
Gastric polyps	1 (2.4)	0 (0)	0.45
Gastric tumor	1 (2.4)	0 (0)	0.45

Abbreviations: QC, concomitant quadruple therapy; TT, triple therapy.

In the intention to treat, the Hp eradication rate was 81% for the patients who received QC versus 72% for those who were treated with TT, with no significant difference between the two (Table 3).

**Table 3:** *Helicobacter pylori* eradication rate in the two groups according to the type of analysis.

	Eradication rate		*p*
	QC *n* (%)	TT *n* (%)	
Intention to treat (*n* = 92)	34/42 (81)	36/50 (72)	0.31
Per protocol (*n* = 84)	31/38 (81.6)	35/46 (76.1)	0.54

Abbreviations: QC, concomitant quadruple therapy; TT, triple therapy.

In multivariate analysis, no predictor of Hp eradication failure, such as age, gender, smoking, overweight, and therapeutic compliance, was found in this study (Table 4).

**Table 4:** Predictors of *Helicobacter pylori* eradication failure in the two treatment protocols.

Studied factors		Eradication failure *n* (%)	Success of eradication *n* (%)	*p*
Age < 50 years	Yes	14/22 (63.7)	52/70 (74.2)	0.33
	No	8/22 (36.3)	18/70 (25.8)	
Female gender	Yes	19/22 (86.4)	51/70 (72.8)	0.25
	No	3/22 (13.6)	19/70 (27.2)	
Presence of comorbidities	Yes	2/22 (9.1)	20/70 (28.5)	0.08
	No	20/22 (90.9)	50/70 (71.5)	
Smoking	Yes	2/22 (9.1)	13/70 (18.5)	0.5
	No	20/22 (90.9)	57 (81.5)	
Ethylism	Yes	0/22 (0)	1/70 (1.4)	>0.99
	No	22/22 (100)	69/70 (98.6)	
Low level of education: (illiterate/primary)	Yes	6/22 (27.2)	23/70 (32.9)	0.62
	No	16/22 (72.8)	47/70 (67.1)	
Geographic region: Rural area	Yes	4/22 (18.1)	13/70 (18.6)	>0.99
	No	18/22 (81.9)	57/70 (81.4)	
Overweight (BMI ≥ 25 kg/m²)	Yes	12/22 (54.5)	42/70 (60)	0.65
	No	10/22 (45.5)	28/70 (40)	
High density of *Helicobacter pylori*	Yes	3/22 (13.6)	7/70 (10)	0.69
	No	19/22 (86.4)	63/70 (90)	
Poor therapeutic compliance	Yes	4/22 (18.1)	4/70 (5.7)	0.09
	No	18/22 (81.9)	66/70 (94.3)	

Abbreviation: BMI, body mass index.

The occurrence of adverse effects was noted in 59.5% of patients in the QC group versus 58% in the TT group, with no significant difference between the two (*p* = 0.88). Treatment adherence was also comparable between the two QC and TT groups (90.5% vs. 92%, *p* = 0.36).

## 4 DISCUSSION

Different countries around the world have conducted studies to estimate the rate of Hp eradication according to the recommended treatment protocol QC versus TT. The results of this work are summarized in Table 5.

**Table 5:** Summary of results of the randomized controlled trials concerning the rate of *Helicobacter pylori* eradication according to the treatment protocol: Concomitant quadruple therapy (QC) versus triple therapy (TT).

Studies	Country/year of study	Number of cases	Duration of treatment (days)		Rate of eradication (%)				*p*‐Value	
					ITT		PP			
			TT	QC	TT	QC	TT	QC	ITT	PP
Ang et al.^13^	Singapour 2015	462	10	10	81.7	83.2	95.4	92.8	0.81	0.66
Molina‐Infante et al.^14^	Spain 2015	777	14	14	81.3	90.4	82.3	93.8		<0.001
Liou et al.^15^	Taiwan 2016	1620	14	10	84	86	88	92		>0.05
Kim et al.^16^	South Korea 2019	1141	7	10	63.9	81.2	71.4	90.6		<0.001
Jha et al.^17^	India 2019	121	14	14	49.3	70.1	58.3	77	0.01	0.02
Alfadhli et al.^18^	Kuwait 2022	603	14	14	58.4	64.6	68	78.5		<0.001
Our study	Tunisia 2020	92	14	14	72	81	76.2	81.6	0.31	0.54

Abbreviations: ITT, intention to treat; PP, per protocol.

According to several guidelines, it is admitted that the optimal limit for the Hp eradication rate should be 90% or greater in adherent patients with susceptible infections, with therapy prescribed empirically.^4^, ^19^, ^20^, ^21^

The data from our study, which showed that the eradication rate was statistically similar between TT and QC, are inconsistent with most other works that have shown that the efficacy of QC is better when compared to that of the TT. The differences between the results of previous studies and this one could be explained by the fact that the rate of antimicrobial resistance to metronidazole in Europe, which is 32%,^1^ and in South Korea,^22^ is significantly lower than that of Tunisia which is estimated at 51.3%.^5^ The concordance between the present results and the two Singaporean^13^ and Taiwanese^15^ studies supports this hypothesis because these countries belong to the Asian continent where antimicrobial resistance to metronidazole is high and close to that of the Tunisian population (44%).^6^

Several predictors of the efficacy of eradication treatment have been reported.^7^, ^23^ Among them, three were the most reported: primary resistance of Hp to antibiotics, degree of treatment adherence, and duration of treatment.

The emergence of primary resistance is currently a preeminent and universal problem with dangerously high levels, mainly for clarithromycin, which is a pillar of anti‐Hp therapy.^6^, ^24^, ^25^ In Tunisia, resistance rates are lower compared with neighboring countries^26^ and relatively stable over time^5^, ^27^ These data are very probably falsely reassuring and should be interpreted with prudence because they were not updated and they included low effective. In addition, Tunisia was ranked among the six countries with the highest consumption of antibiotics in the world.^28^

Therefore, although the study of sensitivity was not carried out in this study, the existence of high rates of primary resistance in Tunisia is very likely. This may explain the low rate of Hp eradication, clearly below 90%, observed in our study for the two protocols.

Finally, eradication regimens should be based on the best locally effective regimen, ideally using individual susceptibility testing, or community antibiotic susceptibility, or antibiotic consumption data and clinical outcome data. The agents available differ in different regions, and this, in part, dictates what regimens are possible.^21^

Among the host‐related predictive factors of eradication failure, adherence is the most important.^8^ Recent studies suggest that adherence to prescribed treatment greater than 90% results in better eradication rates, regardless of the treatment prescribed.^11^, ^29^

### 4.1 Limits of the study

− This is a single‐center study with a limited number of patients.
− Previous exposure of patients to antibiotics that may raise the problem of secondary resistance has not been studied.
− The antibiotic sensitivity testing was not carried out because it was not available in the study center.

## 5 CONCLUSION

In this study, there was no statistically significant difference in the rate of Hp eradication between the concomitant quadruple therapy and the 14‐day triple therapy. These eradication rates are well below 90%, which is the lower limit adopted by several guidelines as the optimal threshold for the effectiveness of anti‐Hp cure. Neither regimen should be used topically because of a likely high rate of primary resistance to clarithromycin.

## AUTHOR CONTRIBUTIONS

**Mohamed Hichem Loghmari**: Conceptualization; methodology; project administration; supervision; validation; writing—original draft; writing—review & editing. **Firas Aissaoui**: Data curation; formal analysis; investigation. **Arwa Guediche**: Formal analysis; supervision. **Wided Bouhlel**: Data curation. **Mejda Zakhama**: Data curation; investigation. **Nabil B. Chaabene**: Investigation; supervision; validation; writing—review & editing. **Amel Rehaiem**: Investigation. **Nouha Ben Abdeljalil**: Investigation. **Manel Njima**: Investigation. **Abdelfetteh Zakhama**: Investigation. **Yosr Kadri**: Investigation. **Maha Mastouri**: Investigation; visualization. **Leila Safer**: Conceptualization; supervision; validation; writing—review & editing.

## CONFLICT OF INTEREST STATEMENT

All fees of publication of this manuscript will be supplied by Medis laboratory. Amoxicillin and esomeprazole, as well as the breath test and the internet platform (Dacima) in which patient's data was stored, were supplied by Medis laboratory. Clarithromycin and metronidazole, as well as its placebo, were supplied by Galpharma laboratory. Medis and Galpharma laboratories had no involvement in study design; collection, analysis, and interpretation of data; writing of the report, and the decision to submit the report for publication.

## TRANSPARENCY STATEMENT

The lead author Mohamed Hichem Loghmari affirms that this manuscript is an honest, accurate, and transparent account of the study being reported; that no important aspects of the study have been omitted; and that any discrepancies from the study as planned (and, if relevant, registered) have been explained.

## Data Availability

Data is available on request from the authors. Raw data were generated at the department of hepato‐gastroenterology in Fattouma Bourguiba University Hospital in Monastir. Derived data supporting the findings of this study are available from the corresponding author (Mohamed Hichem Loghmari) on request. The link to the publicly archived datasets is: https://secure.dacimasoftware.net/medis/View/Login.aspx.
